# Genetic diversity of selected genes that are potentially economically important in feral sheep of New Zealand

**DOI:** 10.1186/1297-9686-42-43

**Published:** 2010-12-21

**Authors:** Grant W McKenzie, Johanna Abbott, Huitong Zhou, Qian Fang, Norma Merrick, Rachel H Forrest, J Richard Sedcole, Jonathan G Hickford

**Affiliations:** 1 Department of Agricultural Science, Faculty of Agriculture and Life Sciences, PO Box 84, Lincoln University, Lincoln 7647, New Zealand; 2 Environment Canterbury, PO Box 345, Christchurch 8140, New Zealand; 3 Faculty of Sport and Health Sciences, Eastern Institute of Technology, Private Bag 1201, Napier, New Zealand

## Abstract

**Background:**

Feral sheep are considered to be a source of genetic variation that has been lost from their domestic counterparts through selection.

**Methods:**

This study investigates variation in the genes *KRTAP1-1*, *KRT33*, *ADRB3 *and *DQA2 *in Merino-like feral sheep populations from New Zealand and its offshore islands. These genes have previously been shown to influence wool, lamb survival and animal health.

**Results:**

All the genes were polymorphic, but no new allele was identified in the feral populations. In some of these populations, allele frequencies differed from those observed in commercial Merino sheep and other breeds found in New Zealand. Heterozygosity levels were comparable to those observed in other studies on feral sheep. Our results suggest that some of the feral populations may have been either inbred or outbred over the duration of their apparent isolation.

**Conclusion:**

The variation described here allows us to draw some conclusions about the likely genetic origin of the populations and selective pressures that may have acted upon them, but they do not appear to be a source of new genetic material, at least for these four genes.

## Background

It is thought that livestock genetic variation has decreased through breed substitution and crossing of local and global breeds [1]. Accordingly, interest in feral populations has increased because they are potential sources of genetic variation that may have been lost in commercial sheep flocks [2,3]. It has been argued that reintroducing genetic variability could enhance production in commercial breeds [4].

New Zealand (NZ) has eleven feral sheep populations either on the mainland, or on offshore islands [5]. The mainland populations originated from farmed sheep [6], while those on offshore islands either originated from farms, or were liberated as a food source for mariners [7]. These populations have been described previously [1,4,6,8-13].

In this study, the level of genetic variation of four genes was determined in order to ascertain whether the isolation of these flocks had preserved greater genetic diversity compared to their commercial counterparts in NZ. These four genes are located on three different chromosomes i.e. *KRTAP1-1 *(chromosome 11; a keratin-associated protein gene that encodes a protein KAP1-1 commonly found in wool), *KRT33 *(chromosome 11; encoding wool keratin K33), *ADRB3 *(chromosome 26; encoding the seven-transmembrane domain beta-3 adrenergic receptor ADRB3) and *DQA2 *(chromosome 20; encoding a class II major histocompatibility complex (MHC) protein DQA2).

Previous studies have reported that variations in the keratin and keratin-associated protein genes, including the ones above, influence many wool properties including fibre diameter [14], staple strength [15], mean staple length [16] and the brightness of wool [16]. Accordingly, given the wide phenotypic variation seen in the wool of feral sheep [6,8], one might expect to see increased variation in these genes.

Neonatal lamb mortality, particularly in Merino sheep, represents a large loss to the NZ sheep industry. Allelic variation in *ADRB3 *has been associated with survival in various sheep breeds [17], thus it might be expected that previously reported or new alleles would be found at a higher frequency in feral populations routinely exposed to harsh environmental conditions.

It has been reported that feral sheep may have an increased resistance to a number of diseases. This resistance could imply that variation in key immune function genes such as the highly polymorphic MHC genes is important, as it plays a role in the immune response to pathogens and parasites [18-21].

Collectively the four genes chosen here cover a variety of different animal traits that could be associated with variation in the ability to survive in remote and potentially more severe environments, and where feed availability was probably reduced relative to farmed sheep.

## Materials and methods

### Sheep and DNA sources

Ten feral flocks and two reference flocks (non-feral) were investigated in this study (Table 1). Genomic DNA from these sheep was obtained from whole blood collected on FTA Classic Cards (Whatman BioScience, Middlesex, UK) following the manufacturer's instructions. Reference flock allele frequencies (see Tables 2 and 3) were sourced from published data [17,22-24] and from NZ commercial sheep DNA samples stored at Lincoln University.

**Table 1:** Origin of sheep populations and sample numbers (N)

Flock type	Flock location		Origin/Breed/Type	N
Feral	Offshore	Arapawa Island I	Australia/Merino/unknown	17
		Arapawa Island II	Australia/Merino/unknown	61
		Chatham Island	Australia/Merinos/Saxon	22
		Pitt Island	Australia/Merinos/Saxon	519
		Campbell Island	Australia/Merino × longwool	105
	Mainland	Woodstock	Australia/Merino/unknown	31
		Hokonui I	Tasmania/Merino/Saxon	12
		Hokonui II	Tasmania/Merino/Saxon	73
		Herbert	Tasmania/Merino/unknown	24
		Mohaka	Unknown/Merino/unknown	14
				
				878
				
				
Domestic reference flocks	Mainland	Merino^1^	New Zealand/Merino/unknown	20 123
		All breeds^2^	Corriedale, Poll Dorset, Suffolk, Borderdale, Coopworth, Dorset Down × Coopworth, Merino × Coopworth, Merino × Polwarth, Merino, Polwarth, Dorset Down and Hampshire, NZ Romney, Awassi, Finnish Landrace and other NZ crossbred sheep	43 737

**Table 2:** Within-population sample sizes (N), number of alleles identified (n) and allele frequencies for *KRTAP1-1*, *KRT33* and *ADRB3*

*KRTAP1-1*										
**Population**	**N**	**n**	***A***	***B***	***C***					

**Arapawa Island I**	14	3	0.43^a^	0.46^a^	0.11^bc^					
**Arapawa Island II**	59	2	-	0.93^c^	0.07^bc^					
**Chatham Island**	22	2	0.18^ab^	0.82^ab^	-					
**Pitt Island**	477	2	-	0.85^b^	0.15^a^					
**Campbell Island**	97	3	0.02^c^	0.82^b^	0.16^a^					
**Woodstock**	28	3	0.29^a^	0.68^ab^	0.04^b^					
**Hokonui I**	11	2	-	0.82^ab^	0.18^bc^					
**Hokonui II**	65	2	-	0.88^b^	0.12^bc^					
**Herbert**	23	2	0.15^ab^	0.85^b^	-					
**Mohaka**	6	2	-	0.83^ab^	0.17^bc^					

**Merino reference flock**	795	3	0.23^a^	0.7^a^	0.07^b^					

**All Breeds reference flock**	309	3	0.06^b^	0.80^b^	0.14^c^					

***KRT33***										

**Population**	**N**	**n**	***A***	***B***	***C***	***D***	***E***			

**Arapawa Island I**	13	5	0.04^a^	0.19^ab^		0.46^ac^	0.19^a^			
**Arapawa Island II**	60	3	-	-	0.40^a^	0.23^a^	0.38^b^			
**Chatham Island**	22	5	0.32^b^	0.07^a^	0.16^bc^	0.23^c^	0.23^a^			
**Pitt Island**	471	4	0.04^a^	-	0.05^c^	0.43^c^	0.48^b^			
**Campbell Island**	92	5	0.02^a^	0.42^b^	0.01^a^	0.10^b^	0.45^b^			
**Woodstock**	30	5	0.07^c^	0.18^c^	0.30^b^	0.33^c^	0.12^a^			
**Hokonui I**	11	4	0.23^bc^	0.36^b^	-	0.23^bc^	0.18^a^			
**Hokonui II**	67	5	0.27^b^	0.28^b^	0.14^b^	0.21^a^	0.10^a^			
**Herbert**	24	5	0.13^bc^	0.35^b^	0.31^b^	0.19^b^	0.02^c^			
**Mohaka**	14	2	-	-	-	0.43^c^	0.57^b^			

**Merino reference flock**	739	5	0.26^b^	0.36^b^	0.19^b^	0.04^b^	0.15^a^			

**All Breeds reference flock**	967	5	0.08^c^	0.04^a^	0.05^c^	0.40^c^	0.43^b^			

***ADRB3***										

**Population**	**N**	**n**	***A^1^***	***B^2^***	***C^3^***	***D^4^***	***E^1^***	***F^2^***	***G^5^***	***H^5^***

**Arapawa Island I**	17	4	0.32^bc^	-	0.35^bc^	-	0.24^bc^	0.09^bc^	-	-
**Arapawa Island II**	60	4	0.17^a^	-	0.04^a^	-	0.39^a^	0.40^a^	-	-
**Chatham Island**	22	4	0.27^bc^	-	0.16^c^	-	0.55^a^	0.02^c^	-	-
**Pitt Island**	499	6	0.20^a^	0.04^a^	0.23^c^	-	0.28^a^	0.25^a^	0.002^a^	-
**Campbell Island**	102	4	0.66^a^	0.17^a^	0.1^c^	-	-	0.01^a^	-	-
**Woodstock**	30	4	0.28^bc^	0.05^bc^	0.18^c^	-	-	0.48^a^	-	-
**Hokonui I**	11	3	0.73^a^	0.23^bc^	-	-	0.05^a^	-	-	-
**Hokonui II**	68	4	0.53^a^	0.29^a^	-	-	0.16^bc^	-	0.01^a^	-
**Herbert**	24	4	0.77^a^	0.04^bc^	0.17^c^	-	-	0.02^b^		-
**Mohaka**	6	3	0.25^bc^	0.08^bc^	-	-	-	0.67^a^	-	-

**Merino reference flock**	4 484	6	0.35^b^	0.02^b^	0.33^b^	0.06	0.20^b^	0.05^b^	-	-

**All Breeds reference flock**	13 420	8	0.37^c^	0.09^c^	0.21^c^	0.02	0.20^c^	0.10^c^	0.01^b^	0.004

^1-5 ^represent the effect of gene on cold survival based on the odd ratios reported in [17]: ^1^good survival; ^2^neutral survival; ^3^below average survival; ^4^poor survival, ^5 ^data insufficient to determine the effect on survival; ^a-c ^allele frequency differences within columns that share no common alphabetic superscripts are significantly different (P < 0.05), while those pair wise comparisons that are not different are represented with the same superscripts; "-" represents alleles or data not available

**Table 3:** Within population sample sizes (N), number of alleles identified (n) and allele frequencies for *DQA2*^1^

*DQA2 *alleles												
**Population**	**N**	**n**	***06023***	***0601***	***08011***	***0901***	***0103***	***1101***	***0102-1601***	***0101-1401***	***1201***	

**Arapawa Island I**	17	8	0.18^a^	-	0.15^a^	-	0.06^a^	0.06^a^	-	-	0.03^a^	
**Arapawa Island II**	61	7	0.02^a^	0.02^a^	-	-	-	-	0.03^a^	0.17^a^	-	
**Chatham Island**	22	6	0.23^a^	-	-	0.05^a^	-	0.09^a^	-	-	-	
**Pitt Island**	519	13	0.07^b^	0.02^b^	-	0.11^b^	0.03^b^	0.02^a^	0.04^a^	0.14^a^	0.11^b^	
**Campbell Island**	105	9	-	0.01^b^	-	-	0.07^a^	0.17^b^	-	0.40^b^	-	
**Woodstock**	31	10	0.03^b^	-	-	-	0.05^a^	0.31^b^	-	-	0.24^b^	
**Hokonui I**	12	6	0.05^a^	-	0.13^a^	-	0.08^a^	-	-	0.63^b^	0.04^b^	
**Hokonui II**	73	9	0.07^a^	0.08^a^	0.21^b^	-	0.12^a^	0.13^b^	-	0.23^a^	0.11^b^	
**Herbert**	24	7	0.06^a^	-	0.35^b^	-	0.02^b^	0.08^a^	-	0.23^a^	-	
**Mohaka**	8	2	-	-	-	-	-	0.94^b^	-	0.06^a^	-	

**Merino reference flock**	20123	-	-	-	-	-	-	-	0.04^a^	-	-	

**All breeds reference flock**	43737	-	0.11^a^	0.04^a^	0.03^a^	0.08^a^	0.10^a^	0.01^a^	0.05^a^	0.15^a^	0.13^b^	

***DQA2 *alleles**												

**Population**			***08012-0201***	***0701-1401***	***0701-1301***	***0401-1501***	***0702-1401***	***0301***	***0501***	***0402-1701***	***0401-1601***	

**Arapawa Island I**			0.32^a^	-	-	-	0.09^bc^	-	0.12^b^	-	-	
**Arapawa Island II**			0.11^a^	-	-	-	0.07^bc^	-	0.58^a^	-	-	
**Chatham Island**			0.27^a^	-	-	-	0.23^a^	-	0.14^b^	-	-	
**Pitt Island**			0.002^c^	-	-	-	0.33^a^	0.001^a^	0.01^a^	0.13^a^	-	
**Campbell Island**			-	-	-	-	0.16^a^	0.17^a^	0.005^a^	0.02^bc^	0.005^a^	
**Woodstock**			0.05^bc^	0.02^a^	0.02^a^	0.23^a^	0.03^bc^	-	-	0.03^bc^	-	
**Hokonui I**			-	-	-	-	0.08^bc^	-	-	-	-	
**Hokonui II**			-	-	-	0.01^c^	0.05^bc^	-	-	-	-	
**Herbert**			0.19^a^	-	-	-	-	-	0.06^b^	-	-	
**Mohaka**			-	-	-	-	-	-	-	-	-	

**Merino Reference flock**			0.03^b^	-	0.001^a^	0.04^b^	0.03^b^	-	-	0.02^b^	0.05^b^	

**All breeds reference flock**			0.003^c^	0.02^a^	0.002^a^	0.03^c^	0.02^c^	0.02^b^	0.06^b^	0.02^c^	0.16^c^	

^1^*DQA2 *nomenclature [24]; ^a-c ^allele frequency differences within columns that share no common alphabetic superscripts are significantly different (P < 0.05), while the pair-wise comparisons that are not different are represented with different superscripts; "-" represents alleles or data not available

### PCR amplification and genotyping

PCR amplifications and genotyping approaches were carried out using previously described methods [17,24-26]

### Data analysis

Allele frequencies, number of alleles, observed heterozygosity (*H_O_*), expected heterozygosity (*H_E _*with a Levene's correction) and coefficient of inbreeding (*F*_IS_) estimates based on the method of Weir and Cockerham [27] were determined using GENEPOP version 4.0.7 [28]. This software was also used to determine deviations from Hardy-Weinberg equilibrium (HWE) using the Exact Test with a Markov Chain Method [29] (10 batches, 5 000 iterations per batch and a dememorization number of 10 000). Corrections for multiple significance tests were performed using Fisher's method and by applying a sequential Bonferroni type correction [29]. *F*_IS _estimates were calculated across all the populations and genes (global *F*_IS_) and for individual populations and genes. Allelic richness, a measure of genetic diversity at a single locus, was determined using FSTAT version 2.9.3 [30] and included rarefaction to correct for sample size variation [31].

Allele frequencies for each feral population were compared to those of Merino sheep sourced from NZ commercial farms [17] and to the combined allele frequencies in breeds commonly found in NZ [17,22-24]. This was undertaken to determine which groups were more closely related to each other based on "distance" measured by the Pearson χ^2^-statistic for each possible pair of breeds and their respective estimated gene frequencies.

## Results

All genes investigated in this study were polymorphic and allele frequencies for each gene varied among the studied flocks (Table 2 and 3). No new allele was identified for any of the genes in any of the sheep typed in this study. All the *KRTAP1-1 *alleles previously described were present in the feral sheep except allele *A *absent in four breeds, including one population from Arapawa Island (Hokonui sheep) and one from Pitt Island (Mohaka sheep), and allele *C *absent in the populations from Herbert Forest and Chatham Island.

Previous studies have reported five *KRT33 *alleles [25], all of which occurred in the feral populations. Alleles *D *and *E *were found in all the populations whereas alleles *A *and *B *were absent in the sheep from Arapawa Island and the Mohaka populations, allele *C *was absent in those from Mohaka and alleles *B *and *C *in those from the Pitt Island and Hokonui, respectively.

Six different *ADRB3 *alleles were detected in the feral sheep. The lowest diversity was observed in the Mohaka population with only three alleles while it was greatest in the sheep from Pitt Island with six alleles. It is interesting to note that alleles *D *and *H*, which occur at relatively low frequencies in other commercial breeds in NZ [17], were absent in all the feral populations. The frequency of allele *G *is low in NZ commercial sheep and was only found at a low frequency in the sheep from Pitt Island and Hokonui.

The distribution of *DQA2 *alleles varied considerably among populations with some alleles completely absent in some populations. The lowest diversity was observed in the sheep from Mohaka with only two *DQA2 *alleles. Conversely, thirteen *DQA2 *alleles were present in the sheep from Pitt Island.

For all four genes, in most cases allele frequencies in the feral populations differed significantly (p ≤ 0.025) from the frequencies in the reference flocks, most of the differences being highly significant (p < 0.001). The following exceptions were found: (1) frequencies of *KRTAP1-1 *alleles of sheep from Chatham Island (p = 0.113), Woodstock (p = 0.434), Herbert (p = 0.055) and Mohaka (p = 0.098) were not significantly different from those of the Merino reference flock, and those of sheep from Mohaka (p = 0.673), Campbell Island (p = 0.084) and Hokonui I (p = 0.454) were not different from those of all breeds and (2) frequencies of *ADRB3 *alleles of sheep from the Arapawa Island I did not differ from those of either reference flock (Merino p = 0.332; All breeds p = 0.771).

Allelic richness, observed (*H_O_*) and expected (*H_E_*) levels of heterozygosity and coefficient of inbreeding (*F*_IS_) are shown in Table 4. On average between 2.03 and 4.86 alleles were detected per polymorphic gene across all the populations. The lowest number of alleles was observed for the *ADRB3 *gene (1.59) in the sheep from Arapawa Island II while the greatest number of alleles was found for *KRT33 *(6.12) in the Hokonui II sheep. Allelic richness was highest for *KRT33 *and lowest for *ADRB3 *in all feral populations except for the Mohaka sheep.

**Table 4:** Allelic richness (r), expected (*H_E_*) and observed (*H_O_*) heterozygosity, *F*_IS_^1 ^values for feral sheep populations of New Zealand

Population	Arapawa Island I				Arapawa Island II				Chatham Island				Pitt Island			
locus	r	*H_O_*	*H_E_*	*F*_IS_	r	*H_O_*	*H_E_*	*F*_IS_	r	*H_O_*	*H_E_*	*F*_IS_	r	*H_O_*	*H_E_*	*F*_IS_
***KRTAP1-1***	3.72	0.71	0.61	-0.18	3.30	0.14	0.13	-0.06	3.17	0.36	0.30	-0.20	4.24	0.23	0.26	0.11*
***KRT33***	5.96	0.69	0.73	0.05	4.00	0.58	0.65	0.11**	5.02	0.81	0.78	-0.05	5.95	0.59	0.58	-0.02
***ADRB3***	2.83	0.71	0.73	0.03	1.59	0.75	0.66	-0.13	1.94	0.59	0.62	0.04	1.87	0.77	0.77	-0.01
***DQA2***	4.26	0.82	0.84	0.02*	2.96	0.66	0.62	-0.06	4.48	0.95	0.81	-0.18	2.83	0.82	0.83	0.00

**Mean**	4.19	0.73	0.73	-	2.96	0.53	0.52	-	3.65	0.68	0.63	-	3.72	0.60	0.61	-

																

**Population**	**Campbell Island**				**Woodstock**				**Hokonui I**				**Hokonui II**			
**locus**	**r**	***H_O_***	***H_E_***	***F*_IS_**	**r**	***H_O_***	***H_E_***	***F*_IS_**	**r**	***H_O_***	***H_E_***	***F*_IS_**	**r**	***H_O_***	***H_E_***	***F*_IS_**

***KRTAP1-1***	2.90	0.06	0.30	0.79**	3.41	0.36	0.47	0.24	2.54	0.36	0.31	-0.18	3.05	0.20	0.21	0.03
***KRT33***	4.71	0.59	0.61	0.04	5.49	0.63	0.76	**0.17**	4.41	1.00	0.77	-0.33	6.12	0.85	0.78	-0.09
***ADRB3***	2.06	0.53	0.51	-0.37	2.38	0.80	0.66	-0.21	1.97	0.55	0.44	-0.26	1.79	0.69	0.61	-0.13
***DQA2***	3.03	0.74	0.76	0.02	4.33	0.94	0.81	-0.16**	3.95	0.67	0.60	-0.11	4.52	0.89	0.86	-0.04

**Mean**	3.18	0.48	0.55	-	3.90	0.68	0.68	-	3.22	0.65	0.53	-	3.87	0.66	0.62	-

																
				
**Population**	**Herbert**				**Mohaka**				**Allele richness averages**							
**locus**	**r**	***H_O_***	***H_E_***	***F*_IS_**	**r**	***H_O_***	***H_E_***	***F*_IS_**								
				
***KRTAP1-1***	2.61	0.30	0.26	-0.16	3.00	0.33	0.30	-0.11	3.19							
***KRT33***	5.04	0.79	0.74	-0.07	1.86	0.57	0.53	-0.09	4.86							
***ADRB3***	1.90	0.46	0.38	-0.20	2.00	0.67	0.53	-0.29	2.03							
***DQA2***	4.03	0.79	0.79	-0.01	2.00	0.14	0.14	-	3.64							
				
**Mean**	3.40	0.59	0.54	-	2.22	0.43	0.38	-								

^1^Significance of *F*_IS _is indicated * *P*< 0.05, ** *P*< 0.01, figure in bold character shows a tendency towards significance (P < 0.10); negative values indicate outbreeding while positive values indicate inbreeding; "-" represents data that could not be obtained

Observed and expected heterozygosity values ranged from a low of 0.06 observed for *KRTAP1-1 *to a high of 1.0 for *KRT33*, and a low of 0.13 for *KRTAP1-1 *and a high of 0.86 for *DQA2*, respectively. Arapawa I sheep had the highest mean estimate for *H_O _*and *H_E _*over all of the genes (0.73 and 0.73, respectively), while Mohaka sheep had the lowest mean estimate for *Ho *and *He *(0.43 and 0.38, respectively). Allele sharing was high between animals originating from Campbell Island and Pitt Island for *KRTAP1-1 *and among the Arapawa II flock of feral sheep for *KRT33 *and lower among the Arapawa I flock for *DQA2*. Finally, allele sharing among sheep from Woodstock was very low for *DQA2*.

## Discussion

This is the first report describing DNA variation in feral sheep from NZ. The genes investigated in this study were chosen because they had previously been shown to influence wool traits [16], cold survival [17] and footrot resistance [20,21]. No new allele was identified for any of the genes in the feral sheep, suggesting that they will not be a source of alternative genetic variability, at least for these genes. The allelic richness and heterozygosity results (observed and expected) are comparable with those presented in previous studies of non-NZ wild sheep populations [32-34].

Although the feral sheep sampled were chosen so that they were representative of their populations, there is no guarantee that the farmers who maintain these populations on the NZ mainland have been able to maintain genetic diversity, especially because the flocks sizes are small compared to the original populations. Allele sharing among four offshore island flocks (Arapawa I and II, Pitt Island and Campbell Island) was significant for one gene but not necessarily for the same gene. Sheep populations from both Pitt and Campbell Islands, have undergone extensive size reduction before being relocated to the mainland and it is surprising that the level of inbreeding is not higher. In contrast, among the mainland Woodstock sheep, many different alleles are detected for *DQA2 *suggesting this flock is outbred, although other loci would need to be typed to confirm this. This is most likely due to the ongoing introduction of new genetic material from other Merino sheep which are typically farmed in areas adjacent to this population.

Sources of genetic variation in the feral sheep populations include founder effects, random drift, balancing selection, genetic bottlenecks, or combinations of these. Each will be discussed below.

Genetic drift may have affected these feral populations [35]. However, in some feral populations allele frequencies were similar to those in commercially farmed Merino sheep. This may not be surprising since both the feral and commercial merino sheep share the same Australian origin, and the two groups have been separated at most by 50 generations.

In some cases, allele frequencies in the feral populations were not "Merino-like" and tended to show greater similarity to allele frequencies in other common farmed sheep in NZ. This provides support for the anecdotal contention that these feral sheep have at times interbred with farmed non-Merino sheep.

There is evidence of genetic differences between groups of sheep on remote yet neighbouring islands. Chatham and Pitt Island sheep are thought to be descendents of the same founding Merino sheep, yet they show quite different allele frequencies for many of the genes studied here. Pitt and Chatham Island feral sheep have distinct wool colours but whether this is a result of the differences in the genes studied cannot be ascertained here.

Founder effects may influence the genetic diversity of feral populations [36]. It is apparent from early farming records that many of these flocks were initiated with 50 or less animals and hence the likelihood of finding rare alleles in the founding individuals might be small. Both *ADRB3 *variants *D *and *H *are rare in farmed NZ sheep [17] and they are absent from the feral populations studied here.

An alternative explanation to the founder effect is that particular *ADRB3 *alleles have been lost in the feral populations because they provide no selective advantage. This is called balancing selection and it reflects the situation where alleles are retained in a population by forms of selection such as heterozygote advantage, frequency-dependent selection [37] or selection varying in space and time that favours some alleles in certain environments [38]. *ADRB3 *alleles *A *and *E *are associated with cold survival, alleles *C *and *F *are linked to cold-related mortality, and allele *D *has a strong association with cold-related mortality and total mortality [17]. The complete absence of *ADRB3 *allele *D *in the feral populations could be due to the fact that these flocks were exposed to cold climatic conditions during lambing and death of lambs carrying the allele.

A number of studies have suggested that feral sheep show few signs of susceptibility to infection by ectoparasites [9,12] and fly strike [39] when compared to other domesticated sheep breeds. The reason why these animals may be more resistant to parasites remains unknown, but may involve genetic variation or reduced/non-exposure to the pathogens.

Charbonnel and Pemberton [40] have suggested that infection with *Teladorsagia circumcincta *imposes a selection pressure in the Soay sheep of the island of Hirta in Scotland, and that this is reflected in the temporal divergence of the MHC genes over a relatively short period between 1988 and 2000. In the context of the results reported here, while the MHC allelic richness is at times low, in the absence of any data or evidence of on-going disease challenge it would be speculative to attempt to draw any conclusions. It should be noted that for *DQA2*, allele sharing was high within one island population but low within the mainland feral population, suggesting that the island population may have undergone some selection pressure.

Allele sharing at *KRT33 *and *KRTAP1-1 *was typically low suggesting the flocks may be outbred. Allele richness was highest for *KRT33 *indicating that the level of genetic diversity has remained quite high in these feral sheep populations. Feral sheep populations have some unique wool characteristics including at times a hairy birth-coat type, which has been shown to offer some advantage in improving lamb survival [41-43], the ability to shed their wool [7], tightly curled wool [12] and various coat colours and markings [8]. The genes responsible for these traits have yet to be identified, but may include some of the genes for keratins and KAPs that constitute wool fibre.

Genetic bottlenecks can cause loss of genetic diversity [44]. Like founder effects, they are largely responsible for the loss of low-frequency alleles and tend to increase the abundance of intermediate- and high-frequency alleles [45]. It is generally admitted that sheep populations from Pitt and Campbell Islands originated from a small number of founding animals that multiplied subsequently. After reaching a size of approximately 4 000 sheep on both islands, genetic bottlenecks most likely occurred, when the majority of the sheep were slaughtered, and small numbers of sheep were transferred to NZ to create the flocks studied here. Thus these island populations may have been subject to both founder and bottleneck effects, but the data presented here does not show any strong evidence in favour of the historically documented bottlenecks and there are no obvious differences in allelic richness between the Pitt and Campbell island populations compared to the other feral sheep populations.

## Competing interests

The authors declare that they have no competing interests.

## Authors' contributions

GM supervised the collection of the genotype data, completed most of the analyses and drafted the manuscript. HZ and QF generated the *DQA2 *genotype data. NM provided the genotype data for the keratin genes used in this study. RF developed the *ADRB3 *genotyping methodology and generated the allele frequency data for the Merino and all breeds reference populations used in this study. She also helped revise this manuscript. JA applied for and was granted the funding that underpinned the collection of blood and data from the owners of these sheep. She designed the study and collected the blood samples from the different sheep populations identified. She was involved in typing *KRTAP1-1 *and assisted draft the manuscript. JRS provided completed parts of the statistical analysis and provided useful discussion on the results obtained from this study. He also assisted in the production of the final manuscript. JH helped develop the project in his capacity as research leader, provided comments on the grant proposal, and drafted the final manuscript. All authors read and approved the final manuscript.
